# Silver Nanoparticle Chains for Ultra-Long-Range Plasmonic Waveguides for Nd^3+^ Fluorescence

**DOI:** 10.3390/nano12234296

**Published:** 2022-12-03

**Authors:** Javier Fernández-Martínez, Sol Carretero-Palacios, Pablo Molina, Jorge Bravo-Abad, Mariola O. Ramírez, Luisa E. Bausá

**Affiliations:** 1 Departamento de Física de Materiales and Instituto de Ciencia de Materiales Nicolás Cabrera, Universidad Autónoma de Madrid, 28049 Madrid, Spain; 2 Departamento de Física Teórica de la Materia Condensada, Universidad Autónoma de Madrid, 28049 Madrid, Spain; 3 Condensed Matter Physics Center (IFIMAC), Universidad Autónoma de Madrid, 28049 Madrid, Spain

**Keywords:** silver nanoparticles, linear chains, plasmonic waveguiding, nanophotonics, rare earth ions, energy propagation

## Abstract

Plasmonic waveguides have been shown to be a promising approach to confine and transport electromagnetic energy beyond the diffraction limit. However, ohmic losses generally prevent their integration at micrometric or millimetric scales. Here, we present a gain-compensated plasmonic waveguide based on the integration of linear chains of Ag nanoparticles on an optically active Nd^3+^-doped solid-state gain medium. By means of dual confocal fluorescence microscopy, we demonstrate long-range optical energy propagation due to the near-field coupling between the plasmonic nanostructures and the Nd^3+^ ions. The subwavelength fluorescence guiding is monitored at distances of around 100 µm from the excitation source for two different emission ranges centered at around 900 nm and 1080 nm. In both cases, the guided fluorescence exhibits a strong polarization dependence, consistent with the polarization behavior of the plasmon resonance supported by the chain. The experimental results are interpreted through numerical simulations in quasi-infinite long chains, which corroborate the propagation features of the Ag nanoparticle chains at both excitation (λ_exc_ = 590 nm) and emission wavelengths. The obtained results exceed by an order of magnitude that of previous reports on electromagnetic energy transport using linear plasmonic chains. The work points out the potential of combining Ag nanoparticle chains with a small interparticle distance (~2 nm) with rare-earth-based optical gain media as ultra-long-range waveguides with extreme light confinement. The results offer new perspectives for the design of integrated hybrid plasmonic–photonic circuits based on rare-earth-activated solid-state platforms.

## 1. Introduction

Plasmonic nanostructures constitute a playground for a plethora of phenomena and interactions in the field of nanophotonics. In particular, arrays of metallic nanoparticles (NPs) have been exploited to induce strong coupling with quantum emitters [1], enlarge the interaction range and strength between emitters [2], generate sub-diffractive laser operation in plasmonic nanolasers [3,4,5,6], or for waveguiding applications below the diffraction limit [7,8,9]. Plasmonic waveguides based on the excitation of either surface plasmon polaritons (SPPs) or localized surface plasmon resonances (LSPRs) on NP arrays in the form of linear chains have been shown to be a promising approach to confine and transport electromagnetic energy at subwavelength scales [9,10,11,12,13]. However, despite the progress achieved so far, sub-diffraction waveguiding at ultra-long distances (several tens of microns) is still limited by the ohmic losses intrinsic to metallic components. To mitigate this drawback, different strategies, including the enhancement of interparticle coupling in linear chains of metallic NPs [14,15,16], or the association of optical gain media with plasmonic arrangements, have been proposed [17,18,19,20,21,22,23]. However, the measured propagation distance is still limited to a few microns, and only a few works deal with the study of the long-range optical energy propagation of coupled emitters to plasmonic arrays [2,8,24]. Part of this limitation stems from the technological challenge associated with producing scalable arrangements with very small interparticle distances, and from their combination with optical gain media [14,23,25,26,27,28,29].

In this work, we report on the optical characterization of gain-compensated plasmonic waveguides based on ultra-long (millimeter-long) linear chains of closely spaced Ag NPs assembled on top of a rare-earth (RE)-doped solid-state medium as an efficient plasmonic–photonic platform for photonic guiding with subwavelength confinement. The fluorescence guiding along the silver chains is monitored by means of dual confocal fluorescence microscopy, which enables the spatial separation of the excitation and emission beams. We find that the photoluminescence of Nd^3+^ ions along the chain can be monitored at distances of at least 100 µm from the excitation source. We also observe a polarization dependence of the optical transport consistent with the near-field coupling between Nd^3+^ ions and the collective longitudinal plasmon mode sustained by the Ag NP chains. Numerical simulations based on the finite-difference time-domain method (FDTD) corroborate the near-field coupling of RE emitters into the plasmonic mode and evidence the efficient sub-diffraction guiding of both excitation (VIS) and Nd^3+^ fluorescence (NIR) over several microns, in agreement with the experimental findings. Our results exceed those of previous reports on electromagnetic energy transport using plasmonic NP chains by around an order of magnitude [14,25,28,29], and they hold promise for applications in scalable integrated circuits, advanced sensing, or information processing. Further, the possibility of subwavelength coupling of the fluorescence of RE ions, which are essential components for the development of useful technologies in a future quantum global internet [30,31,32], open new avenues to develop integrated active photonic circuits operating below the diffraction limit. Finally, we would like to mention that, given the broad extinction spectrum of the plasmonic longitudinal mode of the chains, it is expected that the waveguiding operation can be extensible to a variety of different emitters, as long as their emission overlaps the extinction spectra sustained by the chain. This would allow the use of different RE ions as well as other types of emitters, such as transition metal ions or organic dyes.

## 2. Materials and Methods

### 2.1. Sample Preparation

A Nd^3+^-doped periodically poled LiNbO_3_ crystal (Nd^3+^-doped PPLN) was grown using the off-centered Czochralski technique by adding Nd^3+^ as Nd_2_O_3_ in the melt. See reference [33] for additional details. The chains of Ag NPs were fabricated on top of the Z-cut polished crystal surface following the ferroelectric lithography process [34,35]. Briefly, the crystal was immersed into a 0.01 M AgNO_3_ solution at 50 °C while illuminating the surface with UV light at 253.6 nm (Mercury Pen-Lamp UVP Model 11SC, Analytik Jena US, CA USA) for 10 min. The emission power of the UV lamp was 5400 μWcm^−2^ at a distance of around 1.9 cm. The sample dimensions were 0.1 × 1 × 1 cm. More details on the sample preparation can be found elsewhere [36]. XRD spectra at glazing incidence showing the presence of metallic silver have been previously reported [37].

### 2.2. Optical Characterization

The fluorescence guiding was carried out in a custom-made dual confocal microscope, which allowed the spatial separation of the excitation and emission location. The sample was optically excited with a 590 nm cw laser (Genesis MX STM, Coherent, Inc., Santa Clara, CA, USA) employing a 100× microscope objective (Olympus, Japan) with NA = 0.9. The excitation beam was focused on the surface, where the NP chains were deposited, with a spot size of around 3 μm (Gaussian-like distribution). Nd^3+^ photoluminescence was collected in transmission geometry by a 40× microscope objective (NA = 0.6), coupled to a XYZ piezoelectric stage (translation range of 200 µm; E-727, Physik Instrumente, Germany). This objective scans the sample surface, enabling us to record the photoluminescence (PL) spectra at different points in the sample, i.e., at different distances from the excitation source. By this means, different PL spatial maps were constructed. The detection was carried out by a Peltier-cooled CCD (Synapse, Horiba) connected to a Horiba Micro HR Monochromator (Horiba). A 1 mm diameter pinhole was inserted in the optical path of the collected PL to increase the spatial resolution. See Figure S1 in the Supplementary Materials for a detailed scheme of the experimental setup.

### 2.3. Numerical Calculations

The 3-dimensional (3D) finite-difference time-domain simulations were carried out with a commercial package (Ansys Lumerical R2.2 2021). A simulation box of 8000 × 2000 × 800 nm^3^ containing a single chain of 76 closely spaced spherical Ag NPs of 50 nm size, with a gap distance of 2 nm, was considered in agreement with the experiments. Ag NPs were located at z = 0 nm, along the X direction, at the interface between air and the top surface of a semi-infinite LiNbO_3_ substrate (refractive index n = 2.2). The dielectric function of Ag NPs was fitted using the Drude model. Perfectly matched layer (PML) absorbing boundary conditions (specifically, the stretched coordinate PML type) were applied along the three directions. To achieve convergence, we chose a refined mesh below 1 nm in a volume spanning around the Ag NPs. A single dipole source oriented along the chain axis and embedded into the semi-infinite substrate 3 nm below the Ag NP chain was considered as the illumination source.

The calculated extinction and absorption cross-sections of the chains were obtained by means of the boundary element method [38,39]. In these calculations, we considered a finite set of 15 Ag spherical NPs, whose diameters were randomly distributed between 50 and 70 nm, with an interparticle separation of 2 nm. Increasing the number of NPs in the chain above N = 15 only changes the extinction intensity, and not its spectral shape. Convergence of the results was achieved by using two discretization points per nm at each interface between different materials.

## 3. Results and Discussion

RE ion-doped materials are currently exploited in several optical and photonic devices, including solid-state lasers, solar light harvesting, telecom systems, and quantum information, among others [30,31,32,40,41,42,43]. Moreover, the manipulation and control of their optical properties at the nanoscale provides opportunities to further enlarge their multi-functionality in many different strategic areas. In particular, the association of plasmonic nanostructures with RE-doped crystals has been revealed as an interesting approach, enabling solid-state platforms with emergent functionalities at subwavelength scales. For instance, dual-wavelength laser operation assisted by plasmonic structures in RE-doped crystals and plasmon-induced spatial coherence in RE emitters have been recently demonstrated [44,45]. Herein, the association of plasmonic arrangements with RE ions is further exploited to demonstrate the possibility of guiding the fluorescence of Nd^3+^ ions at ultra-long distances in the subwavelength regime by means of plasmonic chains of Ag NPs.

**Figure 1:**
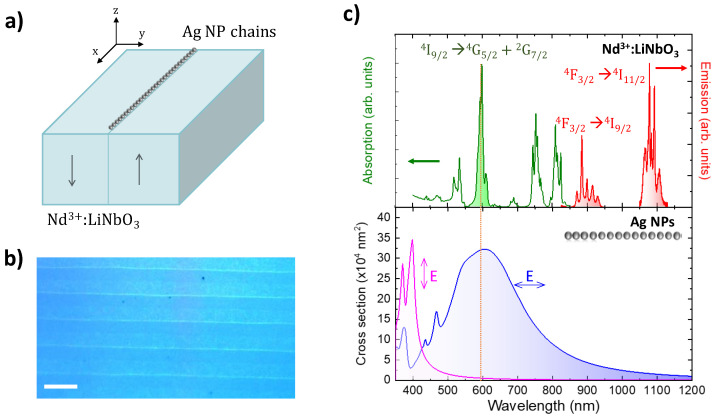
(**a**) Schematic view of the hybrid plasmonic/solid-state system under study. The *xyz* reference system refers to the geometry of the experiment and numerical simulations. (**b**) Optical micrograph of the crystal surface, illustrating the scalability of the fabrication method. The scale bar corresponds to 5 μm. (**c**) Top: absorption (green) and emission (red) spectra of Nd^3+^ ions in LiNbO_3_. For illustrative purposes, the maximum absorption and emission bands have been normalized. Bottom: numerically calculated extinction spectra of Ag NP chains for light polarized parallel (blue) and perpendicular (magenta) to the chain. The dashed orange vertical line indicates the excitation wavelength (λ_exc_ = 590 nm) employed in the experiments.

The hybrid system under study is schematically depicted in Figure 1a. It consists of a *Z*-cut, periodically poled lithium niobate crystal (PPLN), a uniaxial ferroelectric material, homogeneously doped with optically active Nd^3+^ ions. Hence, ferroelectric domain structures of alternating polarity can be used as templates for the domain-selective formation of metallic nanostructures on the crystal surface by a very simple and cost-efficient photo-reduction process known as ferroelectric lithography [34,35,46]. In the case of LiNbO_3_, the high values of the normal electric field component at the domain boundary surfaces can be used to induce the formation of ultra-long chains of closely spaced spherical metallic nanoparticles on the domain boundaries’ surfaces [35,46]. Additionally, LiNbO_3_ offers the possibility of incorporating optically active ions during its crystal growth, which enables the integration of plasmonics and solid-state gain media. Further, a variety of different types of optical waveguides have been demonstrated in this system, including optical amplifiers and lasers [47,48,49,50]. Regarding Nd^3+^, it is the most widely employed active ion in solid-state gain media due to, among others, the large quantum efficiency, the four-level scheme for laser operation, and the numerous absorption bands in the near-infrared and visible spectral regions, which ensure efficient optical pumping [40]. The optical image displayed in Figure 1b illustrates the macroscopic extension of the Ag NP chains formed on the domain walls. The average diameter of the Ag NPs is approximately 50 nm and the interparticle separation is close to 2 nm. The separation distance between the linear chains is close to 3 µm, as defined by the ferroelectric domain width.

The normalized absorption (green) and emission (red) spectra of Nd^3+^ ions in LiNbO_3_ are shown in Figure 1c (upper panel). The analysis of the crystal field transition of Nd^3+^ unambiguously confirms that Nd^3+^ is incorporated into the LiNbO_3_ matrix, in agreement with previous results [51,52]. The absorption bands in the visible and NIR spectral region are associated with the electronic transition from the fundamental ^4^I_9/2_ state to different manifold excited states. Nd^3+^ emission occurs in the NIR spectral region. According to the Dieke diagram, the main emission bands centered at around 900 nm and 1080 nm correspond to the ^4^F_3/2_ → ^4^I_9/2_ and ^4^F_3/2_ → ^4^I_11/2_ electronic transitions of the Nd^3+^ ion, respectively [49]. For the experiments, the excitation was carried out at λ_exc_ = 590 nm (^4^I_9/2_ → ^4^G_5/2_ + ^2^G_7/2_ transition) [53].

The far-field optical response of the fabricated Ag NP chains exhibits a narrow mode polarized perpendicular to the chain at around λ = 400 nm, and a broad longitudinal mode polarized parallel to the chain peaking at λ = 600 nm, which extends from the visible to the NIR (see bottom panel in Figure 1c). The longitudinal plasmonic resonance spectrally overlaps the optical transitions of Nd^3+^, enabling the enhancement of the spontaneous emission of these ions in the vicinities of the metallic NPs [37,39,54].

**Figure 2:**
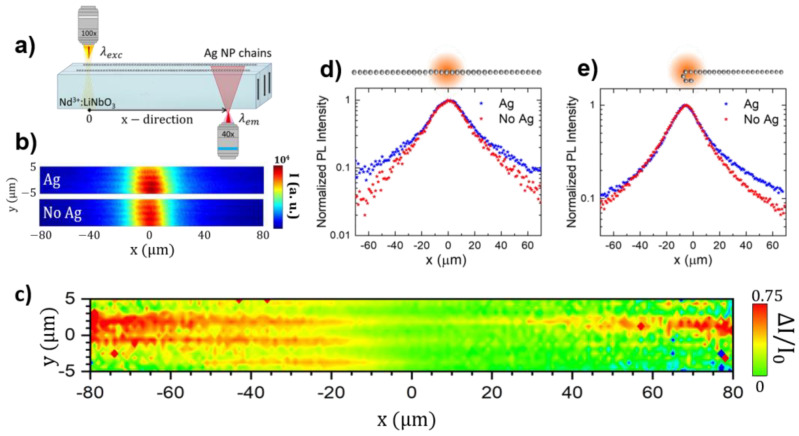
(**a**) Schematics of the experimental set-up. (**b**) Nd^3+^ PL spatial maps obtained after integrating the ^4^F_3/2_ → ^4^I_9/2_ emission (centered at around 900 nm) when exciting on top of a plasmonic chain (Ag) and on the bare surface of the substrate (No Ag). (**c**) Differential emission map obtained from the images displayed in panel (**b**). (**d**) Normalized PL intensity profiles extracted from the Ag and No Ag images along the X direction, where the plasmonic chain is located. The excitation is focused on *x* = 0 μm. (**e**) Normalized PL intensity profiles obtained for a bended plasmonic chain.

The possibility of long-range optical energy propagation and fluorescence guiding along the silver chains is studied by means of dual confocal fluorescence microscopy. Figure 2a shows a schematics of the experimental set-up. The excitation is focused on the surface side of the crystal that supports the chains, and the Nd^3+^ PL is collected in transmission geometry at a certain distance, *x*, from the excitation source. Nd^3+^ PL spatial maps were recorded by fixing the excitation spot on a plasmonic chain (“Ag” hereafter), as well as on the bare surface of the crystal (“No Ag”). Figure 2b displays both Ag and No Ag PL images, obtained after integrating the emitted intensity associated with the ^4^F_3/2_ → ^4^I_9/2_ transition around the emission wavelength λ_em_ = 900 nm [53]. The central spot in the images corresponds to the spatial distribution of the spontaneous emission at the spatial region where the excitation beams is focused. It exhibits a Gaussian-like distribution consistent with the spatial distribution of the laser excitation beam. It should be noted that in the range of excitation power used in our experiments, the emission intensity varies linearly with the excitation intensity. Therefore, the emission profile obtained in the absence of plasmonic chains directly reflects the spatial distribution of the excitation beam and so delimits the spatial extent of the PL signal that is locally collected at the excitation region.

Using these images, to reduce the background PL contribution arising from the bulk substrate and the non-metalized areas excited by the laser beam, we calculated the differential emission map as $\Delta I/I_{0}\equiv \left(I_{Ag}\left(x,y\right)-I_{No\;Ag}\left(x,y\right)\right)/I_{No\;Ag}\left(x,y\right)$. The results are shown in Figure 2c. The NP chains are now clearly identified as linear straight filaments with high values of differential emission, which indeed increases at successively increasing distances away from the excitation spot (located at *x* = 0 μm, *y* = 0 μm coordinates in the image). These growing intensity values, recorded as a function of distance on the Ag NP chains, can be attributed to an active waveguiding effect driven by the coupling of the Nd^3+^ emitters with the longitudinal plasmonic mode of the chain. In other words, the plasmonic chain effectively confines and guides the PL arising from locally excited Nd^3+^ ions and out-couples part of the emitted intensity, re-radiating it to the far field.

To obtain a better insight into the enhancement and propagation of PL along the chains, Figure 2d shows the normalized intensity profiles of the PL intensity extracted from the Ag and No Ag images along the X direction, where the plasmonic chain is located. With this normalization, the local plasmonic enhancement of the emission and excitation can be disregarded, enabling the analysis of long propagating modes along the metallic chain. The comparison between both profiles confirms the different behavior of Nd^3+^ fluorescence in the vicinity of the chains, particularly when the integrated PL intensity is recorded at distances away from the excitation spot. More specifically, at a distance of 60 µm from the excitation region, the total integrated intensity in the vicinity of the plasmonic chain is roughly one order of magnitude larger than that recorded in the bare substrate. This fact evidences the capability of plasmonic waveguides to enhance and propagate the PL arising from locally excited Nd^3+^ ions coupled to the chain. The sub-wavelength fluorescence waveguiding in the hybrid system was also proved in sample regions where the plasmonic chains bent. As shown in Figure 2e, this geometry allows the evaluation of the integrated PL intensity as a function of distance from the excitation spot with and without plasmonic chains in the same line scan. The results show that the increase in the PL intensity only occurs on the right-hand side of the excitation beam where the chain is located, confirming the long-range optical energy propagation of Nd^3+^ emitters coupled to the chain, which extends over tens of microns from the excitation spot due to the excitation of plasmonic modes.

**Figure 3:**
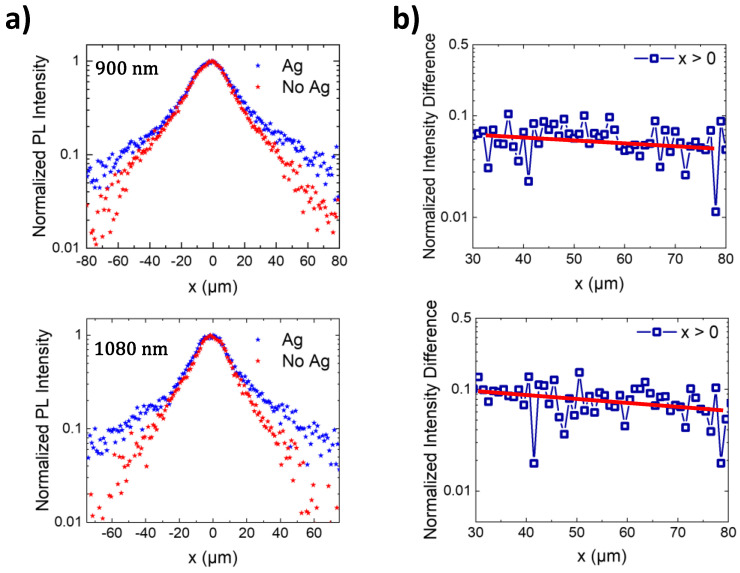
(**a**) Normalized PL intensity profiles associated with the ^4^F_3/2_ → ^4^I_9/2_ (top) and ^4^F_3/2_ → ^4^I_11/2_ (bottom) electronic transitions of Nd^3+^ ions. The excitation is focused at *x* = 0 μm. (**b**) Difference between the normalized PL intensity profiles shown in panel (**a**) obtained at spatial distances far away from the excitation beam (x > 30 μm). Solid red lines are guides for the eye.

The possibility of fluorescence guiding was also observed in the spectral region corresponding to the ^4^F_3/2_ → ^4^I_11/2_ transition at around 1080 nm, a Nd^3+^ transition of interest for lasing [5,36,40,55]. The corresponding PL intensity profiles as a function of the distance from the excitation region are shown in Figure 3a. As observed, the PL propagation distance at this wavelength is similar to that measured at 900 nm. The integrated intensity in the vicinity of the plasmonic chain recorded at a distance of tens of microns from the excitation is one order of magnitude larger than that obtained in the absence of nanostructures. Figure 3b shows the difference between the normalized PL intensity profiles with and without Ag chains at spatial distances far away from the excitation beam at two different wavelengths, 900 nm (top panel) and 1080 nm (bottom panel). The slow decay of the PL over distances of around 50 µm shows the efficient PL propagation at the different spectral regions, in agreement with the spectral overlap of the Nd^3+^ emission and the long tail at the low-energy side of the optical response of the metallic NP chain (see Figure 1c and references [39,54,55]).

**Figure 4:**
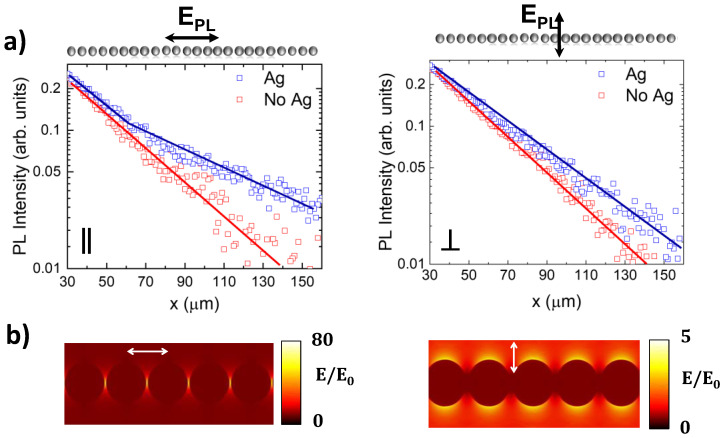
(**a**) Normalized PL intensity profiles associated with the ^4^F_3/2_ → ^4^I_9/2_ transition of Nd^3+^ for fluorescence that is polarized parallel (left) and perpendicular (right) to the axis of the chain. (**b**) Distribution of the near-field amplitudes at the NP midplane computed at λ_em_ = 890 nm for incident plane wave polarized parallel (left) and perpendicular (right) to the chain.

Figure 4a shows the polarized PL intensity profiles recorded for one side of the plasmonic chain at spatial distances far away from the excitation beam. Two main features are observed. First, the long-range energy propagation can be monitored at least 100 µm from the excitation beam. Second, the presence of enhanced subwavelength fluorescence guiding is mainly detected for Nd^3+^ emission polarized parallel to the chain axis, in agreement with the calculated scattering cross-section (Figure 1c) and the near-field amplitude of the plasmonic resonances sustained by the chain (Figure 4b) [39,54,55]. As seen, when light is polarized parallel to the chain, the near field of the longitudinal mode exhibits extreme confinement at the interparticle gaps, leading to large enhancement factors at both the midplane of the NPs and within the substrate [41]. Further, the almost negligible field penetration into the NPs results in low dissipative losses, as desired for subwavelength waveguiding at long propagation distances. In contrast, the transverse mode poorly overlaps the Nd^3+^ emission bands, and its near-electric field amplitude shows a reduced enhancement, which prevents the sub-diffractive optical transport along the chain. Similar results were obtained for the ^4^F_3/2_ → ^4^I_11/2_ electronic transition of Nd^3+^ ions centered at 1080 nm.

**Figure 5:**
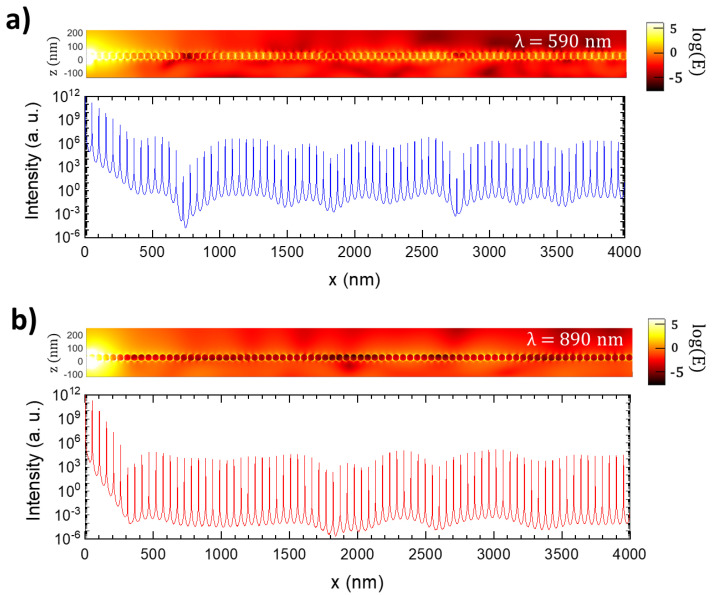
Computed near-field electric field distribution in a Ag NP chain generated by the oscillation of a single electric dipole located at x = 0, z = −3 nm, that emulates an emitting ion at λ = 590 nm (**a**, top) and 890 nm (**b**, top). The dipole oscillates parallel to the *x* axis. Intensity profiles at both wavelengths along the midplane of the NPs (*z* = 25 nm) are shown below in each panel.

Finite-difference time-domain (FDTD) simulations were carried out at two different wavelengths, λ_exc_ = 590 nm and λ_em_ = 890 nm, to corroborate numerically the effect of the plasmonic chain on the Nd^3+^ PL guiding and to obtain additional insights into the subwavelength waveguiding. For this purpose, a single electric dipole oscillating parallel to the chain was located 3 nm under the substrate surface at x = 0 nm, below the gap midpoint between two metallic NPs. This dipole emulates one RE emitter ion, in agreement with the forced electric dipole character of the optical transitions of Nd^3+^ ions in LiNbO_3_ [36]. Figure 5 shows the near-field response of the system under the illumination under such a single dipole. Due to computational limitations, the maximum distance analyzed from the dipole source was 4 μm.

The top panels in Figure 5a,b display the electric field amplitude in the midplane of the NP chain, whereas the bottom panels show the field intensity ($I\propto {\left|E\right|}^{2}$) as a function of distance from the dipole source (located at x = 0). As observed, the electric field propagates along the chain with low losses due to the low penetration of the electric field into the NPs. The propagation is favored at 590 nm, as expected from the spectral response of the longitudinal plasmon chain mode, which peaks at around 600 nm. However, efficient transport at 890 nm is also observed. Indeed, if we neglect the first hundreds of nanometers where the dipole excitation source spreads, the intensity profile at both wavelengths shows an almost flat tendency over distances of at least 4 µm, with intensity maxima associated with the hot spots present in the interparticle gaps. Thus, the results of simulations evidence the capability of ultra-long chains of closely spaced Ag NPs for long-range energy transport with subwavelength confinement. Moreover, the sub-diffractive guiding is obtained in a broad spectral range, which covers both the excitation and emission optical transitions of Nd^3+^ emitters. This fact suggests the possibility of energy exchange between the chain and the substrate in an active waveguide configuration. Additional work is now underway to numerically simulate an active configuration involving Nd^3+^ ions as nano-optical amplifiers in the hybrid system.

In this regard, we have previously shown the presence of the near-field coherent coupling of the Nd^3+^ emitting ions with the plasmonic chain mode, which results in the in-phase de-excitation of the emitters with the plasmonic chain [45]. On the other hand, due to the effective light confinement, the enhancement and coupling of the initial local excitation is also followed by its passive propagation along the chain, which supplies the excitation of the ions in the vicinity of the chain. In this sense, the observation of long-distance emission suggests an effective amplification or compensation effect of the gain material over the plasmonic mode. According to our previous results [45], the emission from these ions excites the plasmon mode that propagates along the chain, which, in turn, induces their oscillation in phase with the chain. Therefore, the continuous exchange of electromagnetic energy between the ions and the chain partially compensates for the optical losses. This underlying mechanism accounts for the long propagation distances observed in our experiments, which surpass, by more than one order of magnitude, previous reports on electromagnetic energy transport using plasmonic NP chains.

## 4. Summary and Conclusions

To summarize, by integrating ultra-long linear chains of closely spaced silver nanoparticles on a rare-earth-doped solid-state platform, we have shown the possibility to enhance and propagate the emission of locally excited Nd^3+^ ions at ultra-long distances in the subwavelength regime. The long-range energy transport primarily originates from both the low dissipative losses displayed by the robust collective longitudinal mode supported by the plasmonic chain and the active waveguiding effect driven by the coherent near-field coupling of the Nd^3+^ emitters with the plasmon mode of the chain. Sub-diffraction fluorescence waveguiding is demonstrated over tens of microns in the technologically relevant NIR spectral range of 900–1100 nm. Full-wave numerical simulations corroborating these observations have been also presented. Remarkably, the obtained propagation range exceeds, by an order of magnitude, that of previous reports on electromagnetic energy transport using plasmonic NP chains, offering an alternative system to metallic strips and nanowires. Considering the ease of the fabrication method and the extraordinary improvement in the subwavelength optical transport, our approach will facilitate the development of novel rare-earth-based integrated optical circuits with potential applications in a diversity of fields, including on-chip quantum photonics, sensing, metrology, and telecom applications.

## Data Availability

Data underlying the results presented in this paper are not publicly available at this time but may be obtained from the authors upon reasonable request.

## Supplementary Materials

The following supporting information can be downloaded at: https://www.mdpi.com/article/10.3390/nano12234296/s1, Figure S1: Schematics of the experimental setup employed in this work.
