# Supplementary material for: Silver Nanoparticle Chains for Ultra-Long-Range Plasmonic Waveguides for Nd3+ Fluorescence
Source: Nanomaterials (Basel). 2022 Dec 3;12(23):4296. doi: 10.3390/nano12234296 (PMC9737231; doi:10.3390/nano12234296)
Supplement: Supplementary file 1 [file nanomaterials-12-04296-s001.zip › nanomaterials-2074620-supplementary.pdf]

nanomaterials

Supplementary Materials

Silver Nanoparticle Chains for Ultra-Long-Range Plasmonic Waveguides for Nd^{3+} Fluorescence

Javier Fernández-Martínez [1], Sol Carretero-Palacios [1], Pablo Molina [1], Jorge Bravo-Abad [2,3], Mariola O. Ramírez [1,3,*] and Luisa E. Bausá [1,3]

Experimental setup: dual confocal microscopy

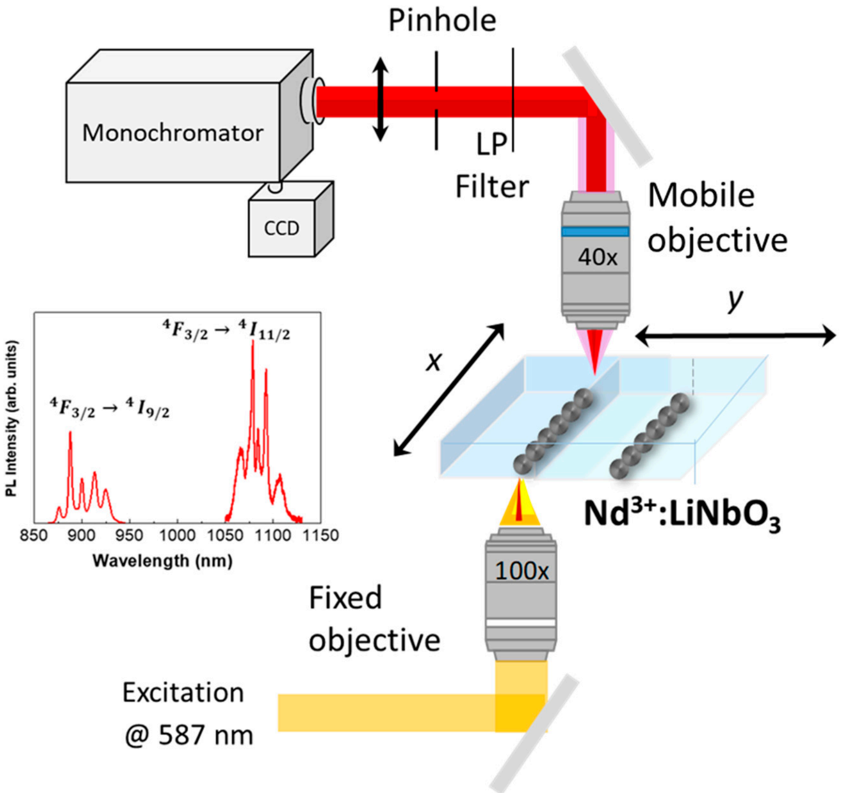

Figure S1. Schematics of the double confocal microscope employed in this study. The sample is excited by a 587 nm cw optically-pumped semiconductor laser (OPSL), focused with a fixed 100x (NA=0.9) microscope objective. The Nd^{3+} photoluminescence (PL) is collected in transmission configuration with a mobile 40x objective (NA=0.6), attached to a XYZ piezoelectric stage. The PL is driven though a lowpass filter, with the aim of supressing the remaining laser, and a 1 mm diameter pinhole to increase the spatial resolution. Detection is carried out by a Peltier-cooled CCD connected to a monochromator. The excitation polarization is set parallel to the axis of the chains by a $\lambda/2$ plate, and a specific polarization of the Nd^{3+} PL can be collected inserting a linear polarizer within its optical path. The inset shows the emission spectrum of Nd^{3+} in $LiNbO_3$ for the $^4F_{3/2} \rightarrow {}^4I_{9/2}$ and $^4F_{3/2} \rightarrow {}^4I_{11/2}$ optical transitions.
